# Clinical therapeutic effects of combined methotrexate and other chemotherapeutic agents in treating children and young patients with osteosarcoma: A protocol for systematic review and meta-analysis

**DOI:** 10.1097/MD.0000000000025564

**Published:** 2021-04-30

**Authors:** Cheng Peng, Zhinan Ren, Jian Zhu, Panke Zhang, Shuyan Cao, Yingjie Hao

**Affiliations:** Department of Orthopedics, the First Affiliated Hospital of Zhengzhou University, Zhengzhou, Henan Province, China.

**Keywords:** adjuvant chemotherapy, meta-analysis, methotrexate, osteosarcoma

## Abstract

**Background:**

Osteosarcoma is one of the most common primary bone tumour in children and young patients, and the third most common among adults. Its main treatment option is currently based on neoadjuvant or adjuvant chemoradiotherapy along with the lesion's surgical resection. The current study's primary aim is to examine the clinical therapeutic impacts of combined methotrexate, along with other chemotherapeutic agents to treat children and young adults suffering from osteosarcoma.

**Methods:**

We will perform a comprehensive literature search in English database (PubMed, EMBASE, Cochran Library CINAHL, and PsycINFO) and Chinese database (Chinese National Knowledge Infrastructure, VIP information database, Chinese Biomedical Database, and WanFang Database) with no language restriction from their inception to the search date. Additionally, two independent authors will screen the works of literature obtained from these databases, obtain information, and examine the risks of data included for the studies’ bias. Furthermore, we intend to employ the *Q* statistics as well as *I*^2^ statistics to calculate heterogeneity among each study's analysis. Accordingly, we will utilize the funnel plots and Egger test to assess the possibility of publication bias where relevant.

**Results:**

The current study aims to provide significant information regarding the clinical therapeutic impacts of combines methotrexate along with other chemotherapeutic agents to treat children and young adults suffering from osteosarcoma.

**Conclusions:**

The present study will generate compelling evidence of combined methotrexate as well as other chemotherapeutic agents for osteosarcoma among children and young adults. Also, it will provide clinical practice suggestions.

**Ethics and dissemination:**

The study is founded upon published data. Therefore, there is no requirement for ethics approval.

**OSF registration number:**

March 26, 2021.osf.io/a23rc. (https://osf.io/a23rc/)

## 1 Introduction

Osteosarcoma is a principal malignant or harmful bone tumour of mesenchymal tissue origin which often affects long bones’ metaphysis.^[1]^ It is among the most common primary bone tumours with an estimated annual frequency of about 400 new cases in the United States, and usually occurs among children and young adults.^[2,3]^ In the clinical realm, osteosarcoma's onset is distinguished by local pain and swelling, as well as occasional joint dysfunction. Some patients suffering from osteosarcoma have been treated for pathological fractures. Even though the degree of malignancy is usually high, some of the confounding symptoms are growth of pain and trauma.^[4]^ Despite multiple attempts with different therapeutic strategies, including adjuvant or neoadjuvant chemoradiotherapy combined with the lesion's surgical resection, survival rates remain significantly unchanged. So far, there are no successful targeted therapies developed for osteosarcoma.^[5–7]^ Accordingly, the five-year survival rate of those experiencing local osteosarcoma tend to reach about 70%. Nevertheless, the long-term survival rate is considered to be around 20% to 30% for metastatic osteosarcoma, while multidrug resistance is a typical issue. ^[2,3,8,9]^ Currently, the majority treatment option for osteosarcoma is founded upon neoadjuvant or adjuvant chemoradiotherapy combined with the lesion's surgical resection.

Chemotherapy regimens generate treatment rates in approximately 70% of patients suffering from localised diseases.^[10]^ In particular, neoadjuvant chemotherapy considerably raises relapse-free survival of patients with non-metastatic diseases and achieves a minimum of about 90% necrosis on the surgically resected tumour.^[11]^ In recent years, methotrexate has become one of the most active drugs, even though it is not manifest whether it is useful for the progress of survival for osteosarcoma among children and younger adults. Different from routine treatment, clinical efficacy is still imperfect. To this end, the current study aims to systematically assess the clinical therapeutic impacts of combined methotrexate along with another chemotherapeutic agents to treat children and younger adults experiencing osteosarcoma.

## 2 Methods

The protocol is registered on the Open Science Framework (OSF, http://osf.io/). Therefore, the present review protocol intends to follow the Preferred Reporting Items for Systematic Review and Meta-Analyses Protocols (PRISMA-P) statement.^[12]^

### 2.1 Eligibility criteria

#### 2.1.1 Types of studies

Randomized controlled trials (RCTs) are essential for comparing the clinical therapeutic effects of treatments, such as methotrexate with treatment without methotrexate to treat children and young adults experiencing osteosarcoma.

#### 2.1.2 Types of participants

This study will include participants (below 21 years) diagnosed with primary osteosarcoma.

#### 2.1.3 Types of interventions

Treatment intends to include methotrexate versus treatment without methotrexate.

#### 2.1.4 Types of outcome

The significant results are the general survival rates and relapse-free survival rates. The minor outcomes are response rate, toxicities, quality of life, and unfavourable events.

### 2.2 Search methods for identification of studies

#### 2.2.1 Search strategy

The search strategy will entail performing a comprehensive literature search among the English database (PubMed, EMBASE, Cochran Library CINAHL, and PsycINFO) and Chinese database (Chinese National Knowledge Infrastructure, VIP information database, Chinese Biomedical Database, and WanFang Database) with no language restriction from their inception to the search date. We will use the following subject heading terms “osteosarcoma∗,” “chemotherapy∗,” “methotrexate∗,” “randomized controlled trial,” “randomised controlled trial,” randomly∗, and RCT∗ along with Boolean operators (AND, OR).

#### 2.2.2 Search strategy

We intend to scan the International Standard Randomized Controlled Trial Number Register (https://www.isrctn.com/) and ClinicalTrials.gov (www.ClinicalTrials.gov) to identify ongoing trials.

### 2.3 Data collection and analysis

#### 2.3.1 Study selection and data extraction

**Figure 1:**
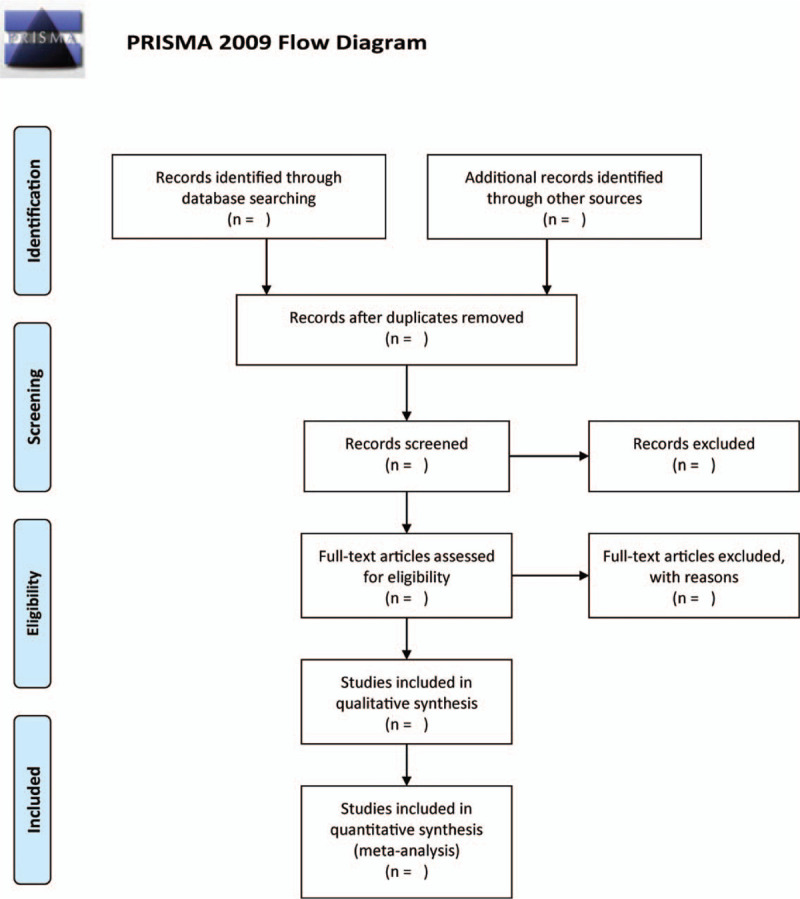
The research flowchart.

We will use two independent authors individually screen titles/abstracts of the search results for potentially relevant studies. The other literature will entail reading the full texts of all possibly relevant studies to sort them out in terms of eligibility. We will also extract the following data using a standardized data extraction form: author's first name, date of publication, country, age, sex ratio, number of participants, intervention and comparison measures, duration of therapy, and outcomes. In case of disagreements, we will discuss them with a third author or a third author's consultation where needful. Figure 1 illustrates the selection process.

#### 2.3.2 Risk of bias assessment for individual studies

We will refer to the Cochrane Collaboration's Handbook recommendations and use this in evaluating the methodological quality of the included RCTs studies.^[13]^ Any disagreements will be discussed with a third author to reach a consensus.

#### 2.3.3 Statistical analysis

We will conduct statistical analysis using the RevMan 5.3 software. We will then analyse the resulting dichotomous data with the relative risk/risk ratio and 95% confidence interval. We will also express data on continuous outcomes as the mean difference or standardised mean difference and 95% confidence interval. We intend to evaluate heterogeneity across studies by the Cochrane *Q* test and quantified by calculating *I*^2^ statistic and defined as substantial heterogeneity if *I*^2^ more than 50%. We will also use the included studies’ heterogeneity, the fixed-effect model, or the random-effects model where needful.^[14,15]^ Then, we will conduct sensitivity analysis to examine our findings’ stability and robustness by either adding or removing studies. Lastly, we plan to employ the funnel plots and Egger test to assess publication bias potential where necessary.

## 3 Discussion

Over the recent past, the RCTs of combined methotrexate along with other chemotherapeutic agents used to treat children and younger patients experiencing osteosarcoma have progressed gradually. Many existent works of literature have supposed that applying the combined methotrexate and other chemotherapeutic agents can have a significant impact on osteosarcoma. Nevertheless, the variation in clinical therapeutic impacts compared with chemotherapy without methotrexate seems to be imprecise. Therefore, the present study seeks to first explore the clinical therapeutic impacts of combined methotrexate along with other chemotherapeutic agents to treat children and young patients experiencing osteosarcoma. The study aims to promote more evidence-based policies and practice towards improving the chemotherapeutic approach of osteosarcoma.

## Author contributions

**Conceptualization:** Cheng Peng, Panke Zhang, Yingjie Hao.

**Data curation:** Cheng Peng, Jian Zhu.

**Formal analysis:** Cheng Peng, Panke Zhang, Shuyan Cao.

**Funding acquisition:** Zhinan Ren.

**Investigation:** Zhinan Ren.

**Methodology:** Zhinan Ren, Panke Zhang.

**Project administration:** Zhinan Ren, Jian Zhu, Shuyan Cao.

**Resources:** Zhinan Ren, Jian Zhu, Panke Zhang, Yingjie Hao.

**Software:** Cheng Peng, Panke Zhang, Shuyan Cao.

**Supervision:** Zhinan Ren.

**Validation:** Cheng Peng, Jian Zhu, Panke Zhang, Shuyan Cao.

**Visualization:** Zhinan Ren, Jian Zhu.

**Writing – original draft:** Cheng Peng.

**Writing – review & editing:** Jian Zhu, Yingjie Hao.
